# Signet ring cell carcinoma of early gastric cancer, is endoscopic treatment really risky?

**DOI:** 10.1097/MD.0000000000007532

**Published:** 2017-08-18

**Authors:** Sun Hyung Kang, Joo Seok Kim, Hee Seok Moon, Eaum Seok Lee, Seok Hyun Kim, Jae Kyu Sung, Byung Seok Lee, Hyun Yong Jeong

**Affiliations:** Department of Internal Medicine, School of Medicine, Chungnam National University, Daejeon, Republic of Korea.

**Keywords:** endoscopic treatment, lymph node, signer ring cell carcinoma, stomach

## Abstract

Signet ring cell carcinoma (SRC) is a poorly differentiated cancer of the stomach. Generally, poorly differentiated cancer is believed to show poor prognosis and aggressive behavior. Recently, however, there is debate on the aggressiveness of SRC in early gastric cancer (EGC). We therefore studied postoperation biopsies to investigate the aggressiveness of SRC in EGC.

We reviewed medical records of patients with EGC who had surgery from January 2011 to December 2015 in a tertiary hospital in Daejeon, South Korea. We evaluated the histologic type, invasion depth, lymphovascular invasion (LVI), and lymph node (LN) metastasis.

A total of 822 EGC lesions from 789 patients were studied. Approximately 498 differentiated cancer, 65 poorly differentiated cancer, 91 SRC, 26 poorly differentiated with SRC, 41 mixed type, 10 medullary carcinoma, and 91 poorly cohesive carcinoma other than SRC were included. LN metastasis was associated with the histologic type of EGC (*P* = .000). Nine percent of differentiated cancer, 21.5% of poorly differentiated cancer, 5.5% of SRC, 11.5% of poor differentiation with SRC, 26.8% of mixed type, 20% of medullary type, and 15.4% of poorly cohesive carcinoma other than SRC showed LN metastasis. The risk of SRC was not higher than well to moderated differentiated cancer (odds ratio [OR] = 0.842, *P* = .768). Risk of LVI was also similar with LN metastasis. Compared with differentiated cancer, OR of SRC was 1.969 (*P* = .172).

Our results show that SRC is not more aggressive than differentiated cancer. SRC may be considered a candidate for endoscopic treatment.

## 1 Introduction

A signet ring cell carcinoma (SRC) of the stomach is a subtype of a poorly cohesive carcinoma (WHO classification).^[1]^ The characteristic ring appearance of an SRC is due to its mucin-rich cytoplasm and crescent-shaped nucleus.^[1]^ According to the Japanese Classification System,^[2]^ SRCs of the stomach are classified as undifferentiated, whereas they are classified as diffuse according to Lauren classification.^[3]^ Unlike other types of gastric adenocarcinomas, the signet ring cells do not adhere to each other due to decreased expression of E-cadherin, which is associated with cell-to-cell adhesion.^[4]^ E-cadherin deficiency leads to migration and invasion of adjacent tissues.^[5]^ Thus, the prognosis of an SRC is considered poor,^[6]^ and surgical resection is generally the treatment of choice. However, a study in South Korea reported a lower rate of lymph node (LN) metastasis in early stage gastric SRCs and suggested that SRCs were possible candidates for minimally invasive surgery.^[7]^ Many follow-up studies reported similar results,^[8–12]^ supporting the possibility that SRCs might be candidates for endoscopic resection. Despite growing evidence in support of endoscopic resection, endoscopic treatment for early stage SRC has not been widely accepted.

Unlike the lymphatic system in the colon, which is located in the submucosal (SM) layer, the lymphatic system of the stomach is located deep in the mucosal layer. As an SRC is easily separated from the main lesion, many physicians are concerned about potential lymphovascular invasion (LVI), LN metastasis, and remnant cancer after endoscopic resection. Thus, surgical treatment remains the treatment of choice for early stage SRC. However, surgical resection causes diverse dietary complications. To improve quality of life, minimally invasive endoscopic resection, especially endoscopic SM dissection (ESD), has been attempted for the treatment of early stage gastric SRCs in South Korea and Japan. Confirming the safety of ESD for early stage gastric SRCs would improve the quality of life of patients and remove the need for invasive surgical treatment. Therefore, a retrospective study was performed to evaluate the safety of ESD for early stage gastric SRCs.

## 2 Materials and methods

### 2.1 Study population

The medical records of patients with early gastric cancer (EGC) who had surgery between January 2011 and December 2015 in a tertiary hospital in Daejeon, South Korea were reviewed. The histological type, depth of invasion, LVI, and LN metastasis were evaluated after surgery. In total, 822 EGC lesions from 789 patients were assessed, and the patients’ medical records, including endoscopy records, pathological reports, radiological reports, and laboratory data, were analyzed. If a patient had multiple EGCs and LN metastasis, LN metastasis was considered to have originated from EGC with deeper invasion or LVI.

Subtotal or total gastrectomy with D1 + α or D2 lymphadenectomy was performed. Histological types of EGC were classified as well to moderately differentiated, poorly differentiated, SRCs, poorly differentiated types with SRC components, mixed types, medullary carcinomas, and poorly cohesive carcinomas other than SRCs. The histological type was determined by the dominant cell type (>50%). The carcinomas were classified by the histological type, gross morphology, size, depth of invasion, LVI, and LN metastasis, and the associations of these factors with LVI and LN metastasis were analyzed. The relative risk of LN metastasis and LVI with each factor was also determined. SM cancer invasion was divided into 3 categories: SM1 cancers were those that invaded the upper one-third of the SM layer, SM3 were those that invaded more than two-thirds of the SM layer, and SM2 cancers were those that invaded between SM1 and SM3. The tumors were classified into 4 categories according to their sizes: smaller than 1 cm, between 1 and 2 cm, between 2 and 3 cm, and larger than 3 cm. The depth of invasion was classified as mucosal (M cancer) and SM (SM1, SM2, and SM3). The local ethics committee agreed to this nonintentional retrospective study exemptions ethical approval and informed consent. This study was conducted in accordance with the principle of the Declaration of Helsinki and International Conference for Harmonization.

### 2.2 Statistical analysis

Statistical analysis was performed using SPSS software, version 18.0 (SPSS, Chicago, IL). A univariate analysis was performed using a chi square test, and Fisher exact test was used to evaluate the causal relationship between the risk of LN metastasis or LVI and other factors (histologic type, size, depth of invasion, gross morphology, etc). A multivariate analysis was performed using a logistic regression analysis to calculate odds ratios (ORs) of LN metastasis and LVI. The accepted level of significance was *P* < .05.

## 3 Results

The study consisted of 822 EGC lesions from 789 patients: 524 patients were men and 432 patients with M cancer were enrolled. Mean age of study population was 61.98 (Table 1). Of those, 498 were well to moderately differentiated (differentiated cancer), and 65 were poorly differentiated. Ninety-one EGC lesions were SRCs, 26 were poorly differentiated with SRCs, 41 were mixed types, 10 were medullary carcinomas, and 91 were poorly cohesive carcinomas other than SRCs. LN metastasis was associated with the histological type, size, LVI, depth of invasion, and lymphadenopathy on computed tomography (CT) scans (*P* = .05). The gross morphology of the EGC, existence of anemia, and location of the lesion were not associated with LN metastasis. LN metastasis occurred in 9% of differentiated cancers, 21.5% of poorly differentiated cancers, 5.5% of SRCs, 11.5% of poorly differentiated types with SRC components, 29.7% of mixed types, 20% of medullary types, and 15.4% of poorly cohesive carcinomas other than SRCs. LN metastasis was present in 1.6% (2/127 cases) of EGC lesions smaller than 1 cm and in 8.9% (28/316 cases) of EGC lesions between 1 and 2 cm. Only 1 case with no LVI showed LN metastasis. According to the CT scans, 27% (23/85 cases) of positive lymphadenopathy cases and 9.6% (71/737 cases) of negative lymphadenopathy cases showed LN metastasis (Table 2).

**Table 1:**
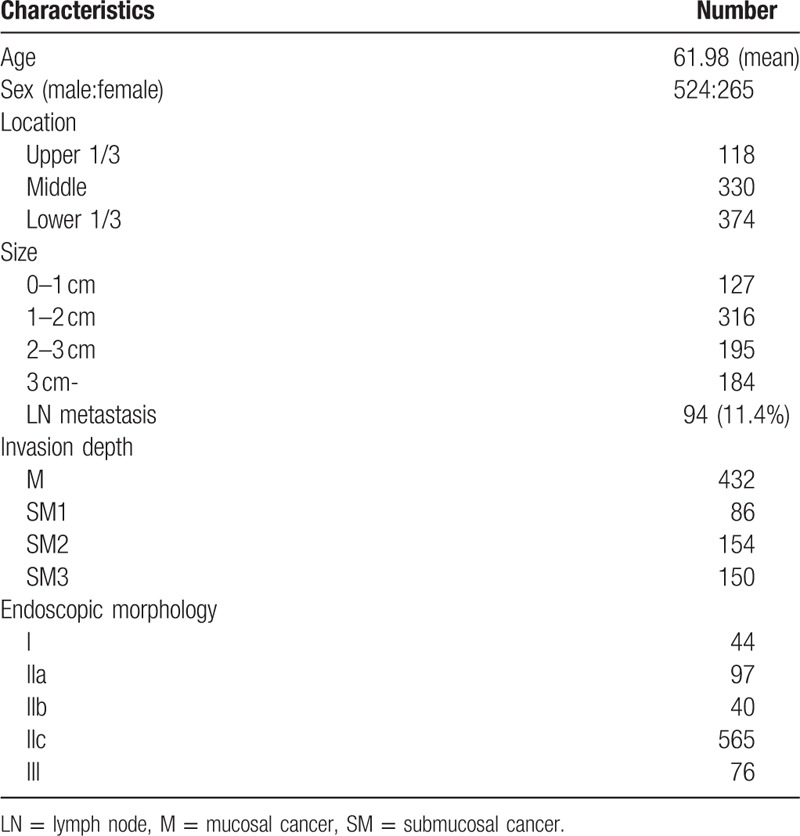
Demographics.

**Table 2:**
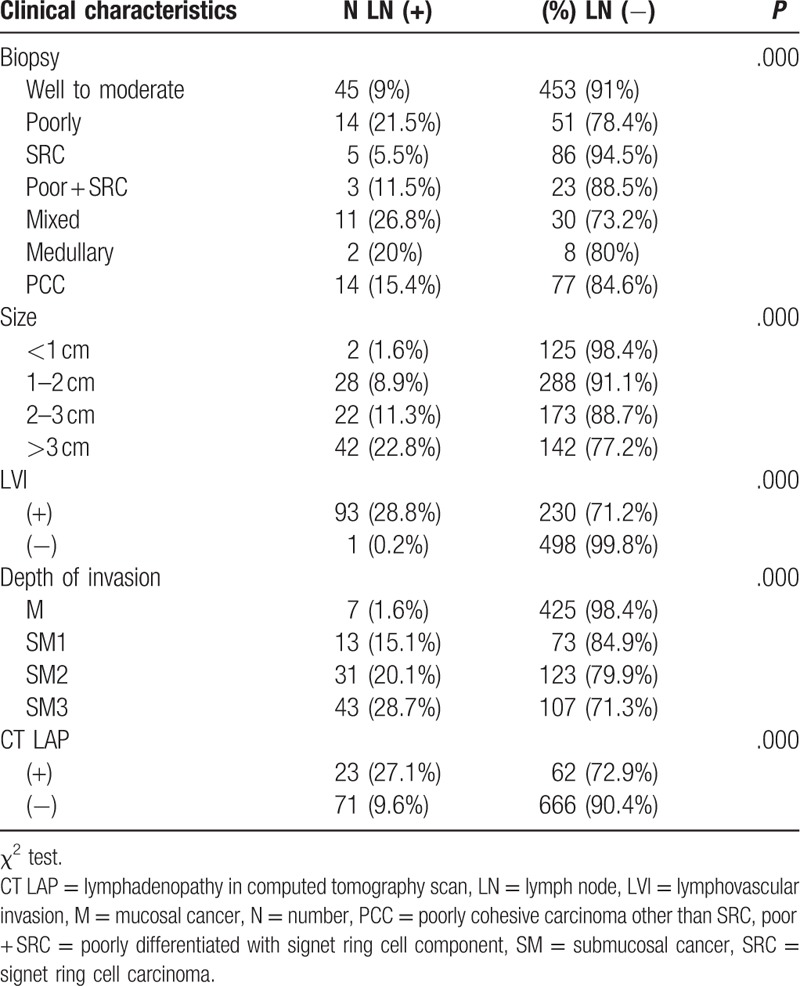
Risk factors of lymph node metastasis.

The risk of LVI was similar to that of LN metastasis. LVI was associated with the histological type, size, depth of invasion, and lymphadenopathy on CT scans (*P* < .05). However, unlike LN metastasis, the existence of anemia was related to LVI (*P* = .023). LVI was present in 37.9% (189/498 cases) of differentiated cancers, 60% (39/65 cases) of poorly differentiated cancers, 24.2% (22/91 cases) of SRCs, 19.2% (5/26 cases) of poorly differentiated types with SRC components, 63.4% (26/41 cases) of mixed types, 50% (5/10 cases) of medullary types, and 40.6% (37/91 cases) of poorly cohesive carcinomas other than SRC. LVI was present in 14 of 432 (3.2%) patients with M cancer, 54.6% (47/86 cases) of patients with SM1 cancer, 84.4% (130/154) of patients with SM2 cancer, and 88% (132/150 cases) of patients with SM3 cancer (Table 3).

**Table 3:**
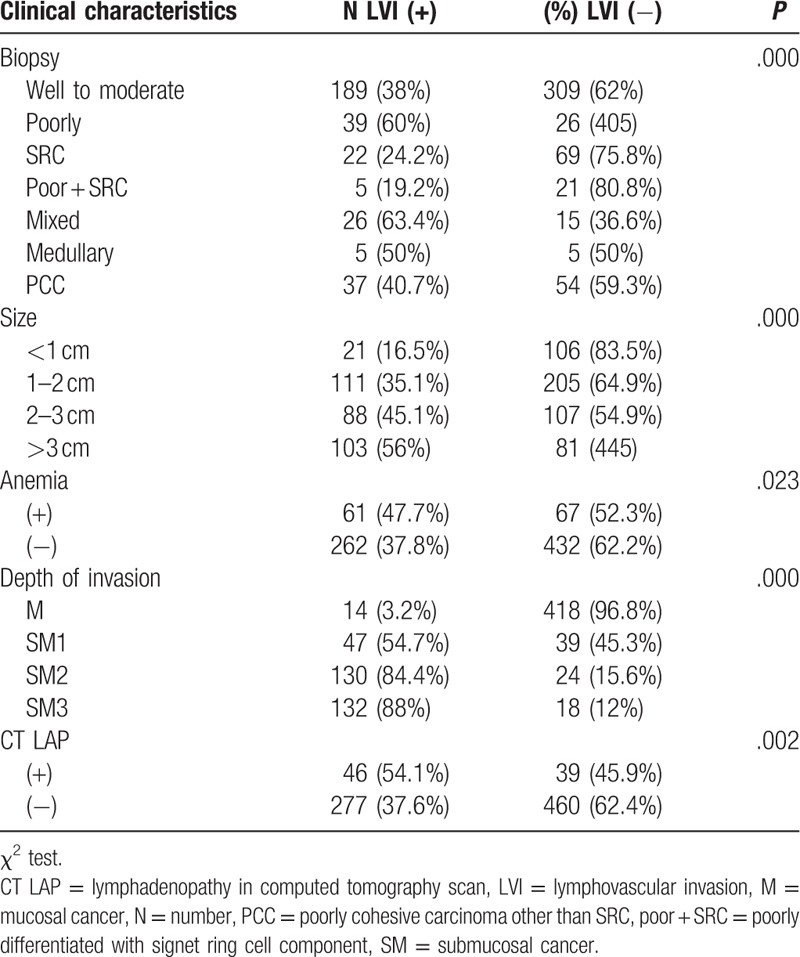
Risk factors for LVI.

In the multivariate analysis, LN metastasis was associated with tumoral size, LVI, and lymphadenopathy on CT scans. As compared to EGC lesions smaller than 1 cm, the OR for LN metastasis was 2.663 for EGC lesions between 1 and 2 cm (*P* = .222), and it was 2.835 for EGC lesions between 2 and 3 cm (*P* = .197). Only EGC lesions larger than 3 cm showed a statistically significant OR (5.782, *P* = .026). Compared to patients with no LVI, the OR for LN metastasis in patients with LVI was 290.7 (*P* = .000). In cases of positive lymphadenopathy on CT scans, the OR was 2.572 (*P* = .008). In the logistic regression, the histological type and depth of invasion did not show a significant difference (Table 4).

**Table 4:**
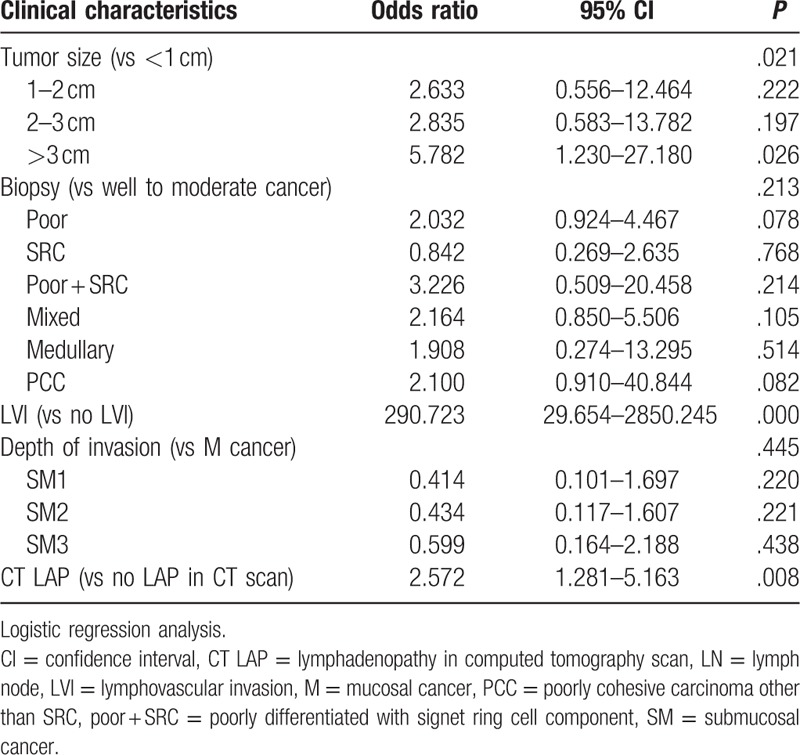
Multivariate analysis of risk factor of lymph node metastasis.

LVI was related to the histological type, size, and depth of invasion in the multivariate analysis (Table 5). Compared to differentiated carcinomas, the ORs for poorly differentiated carcinomas, mixed types, and poorly cohesive carcinomas other than SRCs were significant (*P* = .008 and .014). The OR for SRCs was 1.969 (0.745–5.206, *P* = .172). The existence of anemia and lymphadenopathy on a CT scan were not associated with LVI in the logistic regression.

**Table 5:**
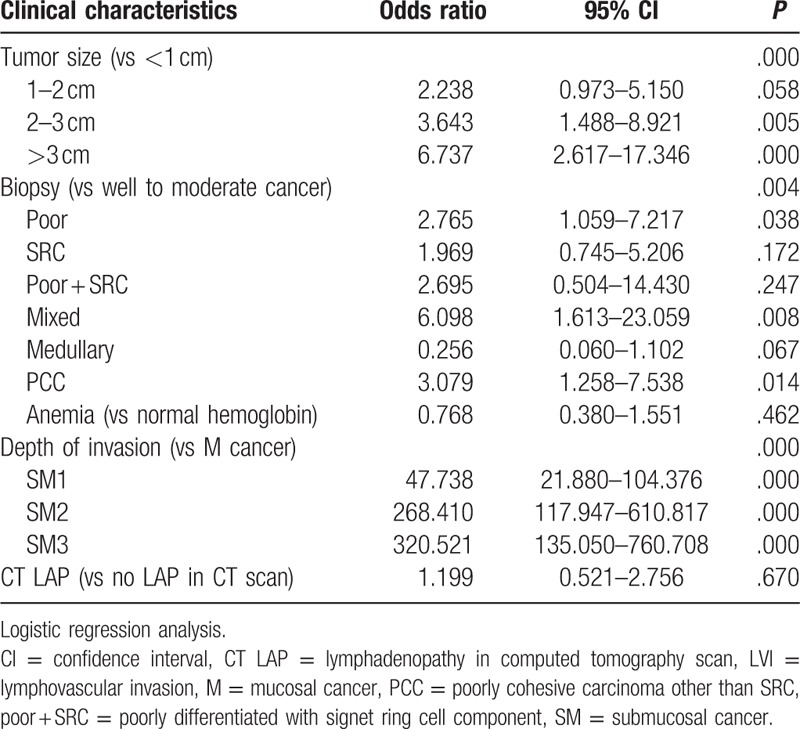
Multivariate analysis of risk factor of lymphovascular invasion.

In 91 cases with SRCs, only 5 cases showed LN metastasis. All the lesions in the patients with LN metastasis were greater than 3 cm. LN metastasis was present in 1.5% (1/66 cases) of M cancer with SRCs, 20% (2/10 cases) of SM2 cancers, and 28.6% (2/7 cases) of SM3 cancers. Eight cases of SM1 cancer with SRCs showed no LN metastasis (Table 6). Six of 253 cases (2.4%) of EGC lesions smaller than 1 cm with differentiated cancer and 2 of 81 cases (2.5%) of M cancer with differentiated cancer showed LN metastasis. Compared with differentiated carcinomas, SRCs did not exhibit aggressive behavior (i.e., LN metastasis and LVI).

**Table 6:**
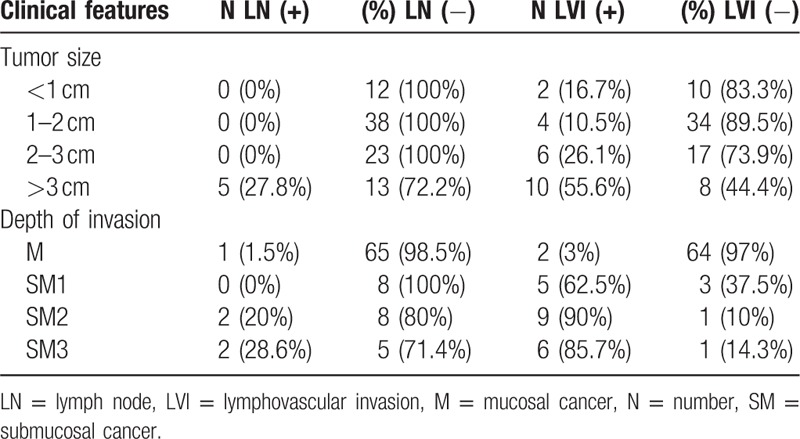
Clinical features of signet ring cell carcinoma.

Follow-up observation of 767 patients’ results showed death in 20 patients, and cancer recurrence occurred in 7 patients (0.9%). Only 2 cases of death were related to cancer recurrence. Other causes included different cancer (6 patients), pneumonia (4 patients), cerebrovascular accidents (3 patients), sudden cardiac death (2 patients), sepsis (1 patient), suicide (1 patient), and postgastrectomy complications (1 patient). Cancer recurrence was related to histology, LN metastasis, and LVI (*P* < .05, χ^2^ test.). Three cases of differentiated cancer (3/485, 0.6%), 1 case of poorly differentiated cancer (1/61, 1.6%), and 3 cases of mixed histology (3/41, 7.3%) showed cancer recurrence. One patient with mixed histology showed metachronous recurrence at the remnant stomach, and 1 patient with differentiated cancer showed nodal and hepatic metastasis. Other 5 cases were nodal metastases.

## 4 Discussion

Previous studies of the prognosis of SRCs reported conflicting results.^[5–10,13–21]^ Several studies strongly suggested a poor prognosis in cases of SRC, including a French study, which also suggested that the prognostic predictors in SRC differed from those in non-SRC.^[13]^ According to the French study, SRC histology was a poor prognostic factor.^[13]^ Several case reports also expressed concerns about the risk of SRCs in EGC. Kobayashi reported 39 cases of overt bone metastasis in patients with EGC, most of whom had SRCs and poorly differentiated carcinomas.^[14]^ Our team also reported overt bone marrow metastasis from small SRCs in EGC.^[15]^ Another French study also showed a poor prognosis in SRC of the stomach, although that study had several limitations,^[16]^ including a relatively small number of patients (n = 215) and a low number of EGC cases (only 20% of the total patient population in the study).

Recent studies of the prognosis in SRC cases reported a favorable prognosis in EGC but a poor or similar prognosis for SRC in advanced gastric cancer.^[17–21]^ Many studies reported less LN metastasis and a favorable prognosis for SRC in EGC.^[5–10,17,19–21]^ In those studies, SRCs which is confined to mucosal layer without LVI and below 2 cm were mostly free from LN metastasis. The risk factors for LN metastasis in those studies were the depth of invasion, LVI, and tumor's size. SRC histology itself was not a risk factor of LN metastasis. The results of the present study were in accordance with those in the literature, as shown by the univariate analysis. Although LN metastasis was most prevalent in the mixed types in the present study, this finding was not statistically significant in the multivariate analysis. The findings of the present study are also is in line with those of a previous Japanese study, which suggested that undifferentiated types and predominantly mixed types of gastric cancer with SM invasion were risk factors for LN metastasis.^[22]^ In addition, a Korean study reported that EGC lesions with mixed SRC histology showed aggressive behavior.^[23]^ In that study, SRC was associated with M cancer and lower LN metastasis, but SRCs with mixed histology showed more SM invasion, larger size, and higher LN metastasis.^[23]^ The findings of these 2 studies and the results of the present study strongly suggest that mixed histology is associated with the risk of invasion and metastasis. Follow-up data suggest that mixed histology was related to cancer recurrence after gastrectomy. No recurrence occurred in patients with SRC.

LVI is an important determinant of the possibility of endoscopic treatment. As mentioned above, the lymphatic system of the stomach is located in the deep mucosal layer, and many physicians are concerned about potential LVI because an SRC is easily separated from the main lesion. The literature on the risk factors for LVI is scarce. In the present study, according to the univariate analysis, LVI was associated with histology, tumoral size, depth of invasion, existence of anemia, and lymphadenopathy on a CT scan. Poorly differentiated types with SRC components showed the lowest LVI, and SRCs exhibited the second lowest LVI. As shown by the logistic regression analysis, compared with differentiated carcinomas, the OR of SRC was 1.969 (*P* = .172). Both the tumoral size and depth of invasion were risk factors for LVI in the multivariate analysis. Mixed histology showed the highest OR (6.098, *P* = .008).

In a Korean study of 448 patients with EGC with SRCs,^[24]^ LN metastasis was present in 10.7% of cases, and LVI was present in 5.6% of cases. Six SRCs less than 2 cm in EGC were associated with LN metastasis, with the smallest SRC being 0.9 cm. In the same study, 5.9% of M cancer in SRC cases showed LN metastasis, which was similar to that observed for differentiated types. The authors recommended gastrectomy for SRCs in EGC because LN metastases from small SRCs were not uncommon. In the present study, SRCs smaller than 3 cm showed no LN metastasis, but 2 cases of M cancer and 2 cases of EGC lesions smaller than 1 cm showed LVI (16.7%). Small or mucosal SRCs may have LVI. In this respect, determining ESD indication for SRCs in cases of EGC should be performed very carefully.

There are several barriers to ESD for SRCs in cases of EGC. The first problem relates to the accurate estimation of the size and margin of the lesions. One study of endoscopic treatment for SRCs in EGC reported underestimations of 30.2% in lesional sizes.^[25]^ In that study, an EGC lesion larger than 2 cm was considered a risk factor for underestimation. However, most studies of the prognosis of SRCs in EGC have been based on data from pathological findings rather than endoscopic data.^[5–10,13–21]^ When determining endoscopic treatment, endoscopist should be aware that the actual lesion could be larger than endoscopic measurements and that it may be risky to treat EGC lesions larger than 2 cm endoscopically. The second problem is the oncological safety of endoscopic treatment. LN dissection is impossible with endoscopic treatment. In some cases, SRCs in EGC may spread to distant organs.^[14,15]^ However, several studies of the outcome of endoscopic treatment for EGC lesions with undifferentiated or poorly differentiated histology, including SRCs, showed no distant metastasis and little LN recurrence.^[26–29]^ In one study in Japan, recurrence in EGC after surgical resection was not associated with the histology of the lesion.^[30]^ The third and final problem is the low complete resection rate. In studies, curative resection was 50% to 60% in cases of undifferentiated histology, and lateral margin involvement was common in SRCs.^[26–29]^ Endoscopic treatment for SRCs in EGC can be increased only after these problems are overcome.

The present study has several limitations. First, it was a retrospective study based on medical records. Second, it consisted of a relatively small number of patients enrolled in a single center. Finally, EGC treated with endoscopic treatment was not evaluated. Approximately 200 to 250 patients with EGC receive endoscopic treatment in our center each year. LN metastasis in EGC lesions smaller than 2 cm with differentiated histology is rare. LN metastasis from differentiated EGC lesions may be overestimated because the data were based on pathological findings after surgical resection. However, as 253 cases of mucosal cancer with differentiated histology were included in the present study, we expect only a small gap between present data and real world.

There are also several strong points in the present study. Unlike other studies, the lesions were classified into a detailed category of histological types. SRCs were distinguished from other poorly cohesive carcinomas. Poorly cohesive carcinoma other than SRC showed more aggressive behavior in our study. We also found risk factors for LVI. As mentioned above, LVI is a very important factor in endoscopic treatment for SRC.

In conclusion, endoscopic resection may be considered as a treatment option for small mucosal gastric SRCs. However, due to limited studies, it should only be performed under strict indications. Also, additional researches are necessary to assess the safety of endoscopic resection for SRC.
